# Hypoxia Enhances Endothelial Intercellular Adhesion Molecule 1 Protein Level Through Upregulation of Arginase Type II and Mitochondrial Oxidative Stress

**DOI:** 10.3389/fphys.2019.01003

**Published:** 2019-08-14

**Authors:** Xiujie Liang, Prakash Arullampalam, Zhihong Yang, Xiu-Fen Ming

**Affiliations:** Laboratory of Cardiovascular and Aging Research, Medicine Section, Department of Endocrinology, Metabolism, and Cardiovascular System, Faculty of Science and Medicine, University of Fribourg, Fribourg, Switzerland

**Keywords:** arginase II, endothelium, hypoxia, intercellular adhesion molecule-1, mitochondrial superoxide

## Abstract

Hypoxia plays a crucial role in the pathogenesis of cardiovascular diseases. Mitochondrial enzyme arginase type II (Arg-II) is reported to lead to endothelial dysfunction and enhance the expression of endothelial inflammatory adhesion molecules such as intercellular adhesion molecule-1 (ICAM-1) and vascular cell adhesion molecule-1 (VCAM-1). In this study, we investigate the role of Arg-II in hypoxia-induced endothelial activation and the potential underlying mechanisms. Exposure of the human endothelial cells to hypoxia induced a time-dependent increase in Arg-II, HIF1α, HIF2α, and ICAM-1 protein level, whereas no change in the protein level of VCAM-1 and E-selectin was observed. Similar effects were obtained in cells treated with a **hypoxia mimetic** Dimethyloxaloylglycine (**DMOG**). Silencing HIF1α, but not HIF2α, reversed hypoxia-induced upregulation of Arg-II. Moreover, silencing Arg-II prevented the ICAM-1 upregulation induced by hypoxia or DMOG. Furthermore, the endothelial cells incubated under hypoxic condition or treated with DMOG or hypoxia enhanced monocyte adhesion, which was inhibited by silencing Arg-II. Lastly, silencing Arg-II prevented hypoxia-induced mitochondrial superoxide production in endothelial cells, and hypoxia-induced ICAM-1 upregulation was reversed by mitochondrial electron transport inhibitor rotenone. These data demonstrate that hypoxia enhances ICAM-1 protein level and monocyte-endothelial interaction through HIF1α-mediated increase in Arg-II protein level on leading to increased mitochondrial reactive oxygen species production. These effects of hypoxia on endothelial cells may play a key role in cardiovascular diseases. Our results suggest that Arg-II could be a promising therapeutic target to prevent hypoxia-induced vascular damage/dysfunction.

## Introduction

Chronic hypoxia plays a key role in the pathophysiology of cardiovascular diseases including hypertension, atherosclerosis, and heart failure (Hulten and Levin, 2009; Bosc et al., 2010; Abe et al., 2017). Hypoxia-inducible factors (HIFs) are well-characterized master regulators combating hypoxia-induced tissue injury by upregulating expression of many genes including erythropoietin, VEGF and glycolytic enzymes, which leads to compensatory increase in the oxygen supply and energy production. However, there is increasing evidence that HIFs also play a key role in the pathophysiology of cardiovascular dysfunction through reducing endothelium-derived protective nitric oxide (NO) (Cowburn et al., 2016), promoting vascular inflammation (Williams and Scharf, 2007; Ben-Shoshan et al., 2009), senescence (Gaspar et al., 2017), and apoptosis (Lee et al., 2005), which leads to vascular diseases. Dissecting HIF-regulated pathways governing the beneficial and detrimental effects would thus shed light for specific targeting of the later pathways while preserving the former.

Arginase is an enzyme metabolizing L-arginine to L-ornithine and urea (Yang and Ming, 2013). There are two isoforms, namely arginase-I (Arg-I) and arginase-II (Arg-II). Arg-I is mainly expressed in hepatocytes and Arg-II in the kidney. Arg-I is located in the cytosol, whereas Arg-II is in the mitochondria. Notably, Arg-II is the main isoform that is inducible in human endothelial cells (Yang and Ming, 2013). Genome-wide analysis of HIF1-binding sites by ChIP-seq in endothelial cells identified that Arg-II gene intron 2 has HIF1-binding regions (Mimura et al., 2012). Studies show that Arg-II is indeed upregulated in response to hypoxia in endothelial cells, which contributes to hypoxia-triggered reduction in NO generation and hypertension (Krotova et al., 2010; Prieto et al., 2011; Cowburn et al., 2016; Pandey et al., 2018). This hypoxia-induced upregulation of Arg-II has been shown to be mediated through HIF2α (Krotova et al., 2010; Cowburn et al., 2016). Moreover, Arg-II was reported to be upregulated by hypoxia in pulmonary artery VSMC resulting in proliferation of the cells (Xue et al., 2017). These provide compelling evidence that Arg-II is a HIF-inducible gene in regulating endothelial NO production and VSMC proliferation under hypoxic condition. It has been shown that endothelial expression of adhesion molecules including vascular cell adhesion molecule-1 (VCAM-1) and intercellular adhesion molecule-1 (ICAM-1) is upregulated by both HIF1α and HIF2α (Ben-Shoshan et al., 2009). However, a role of Arg-II in endothelial activation regulated by HIFs remains unknown.

Compelling evidence including our own has been provided in recent years for an important role of Arg-II, the dominant isoenzyme in human and mouse vasculature, in cardiovascular diseases (Yang and Ming, 2014). It is now well recognized that an enhanced activity and/or expression of Arg-II in endothelial cells compete(s) with eNOS for L-arginine, resulting in eNOS-uncoupling, whereby eNOS produces superoxide anion instead of NO (Yepuri et al., 2012). This mechanism plays an important role in endothelial dysfunction under pathological conditions such as atherosclerosis, type-II diabetes as well as in aging (Yepuri et al., 2012; Xia et al., 2017). Moreover, we have recently demonstrated that Arg-II plays an important role in promoting mitochondrial dysfunction in senescent and apoptotic vascular cells, contributing to atherosclerotic vulnerability phenotype in a mouse model (Xiong et al., 2013). Elevated Arg-II in macrophages in obese mice promotes macrophage pro-inflammatory responses in insulin resistance and atherosclerosis (Ming et al., 2012).

Given the promoting effects of Arg-II on mitochondrial dysfunction and endothelial activation as well as the enhanced Arg-II expression induced by HIFs, we hypothesize that hypoxia-HIF-induced Arg-II in endothelial cells promotes endothelial activation through enhanced mitochondrial oxidative stress.

## Materials and Methods

### Materials

Reagents were purchased or obtained from the following sources: rabbit antibodies against Arg-II (sc-20151) and E-selectin (sc-14011) and mouse antibody ICAM-1 (sc-8439) were from Santa Cruz Biotech (Dallas, USA); mouse antibodies against tubulin (T5168) and β-actin (A5441) and Dimethyloxaloylglycine (DMOG) were from Sigma (St. Louis, Missouri, USA); anti-VCAM-1 (12367S) was from Cell Signaling Technology (Danvers, USA); mouse antibody against HIF1α (610958) was from BD Biosciences (New Jersey, USA); rabbit antibody against HIF2α (PAB12124) was from Abnova (Taipei, Taiwan); Alexa fluor 680 conjugated anti-mouse IgG (A21057), MitoSox, and Hoechst 33342 were from Invitrogen/Thermo Fisher Scientific (Waltham, MA USA); IRDye 800-conjugated anti-rabbit IgG (926-32211) was from LI-COR Bioconcept (Lincoln, USA); endothelial cell growth supplement (ECGS) pack was from PromoCell GmbH (Heidelberg, Germany); and all cell culture media and materials were from Gibco/Thermo Fisher Scientific (Waltham, MA USA).

### Recombinant Adenovirus

The recombinant adenovirus (rAd) expressing shRNA targeting human Arg-II driven by the U6 promoter (rAd/U6-hArg-II^shRNA^) and control rAd expressing shRNA targeting LacZ (rAd/U6-LacZ^shRNA^) were generated as described previously (Yepuri et al., 2012). Generation of rAd expressing shRNA targeting human HIF1α and HIF2α driven by the U6 promoter (rAd/U6-hHIF1α^shRNA^ and rAd/U6-hHIF2α^shRNA^, respectively) was carried out with the Gateway Technology. The targeting sequences are in boldface below (only the sense strand is shown): **GCCGAGGAAGAACTATGAACA** for human HIF1α; **CGACCTGAAGATTGAAGTGAT** for human HIF2α.

### Cell Culture and Adenoviral Transduction of the Cells

Preparation and culture of human umbilical vein endothelial cells (HUVECs) as well as the transduction of HUVECs by recombinant adenovirus were performed as described previously (Yepuri et al., 2012). Hypoxic conditions were achieved by placing the culture dishes or plates in a **Coy** *In Vitro* **Hypoxic Cabinet** System (The Coy Laboratory Products, Grass Lake, MI USA) at 0.2% O_2_ with 5% CO_2_ and N_2_ as balance.

### Immunoblotting

Cell lysate preparation, SDS-PAGE and immunoblotting, antibody incubation, and signal detection were conducted as previously described (Ming et al., 2012). Quantification of the signals was performed using Li-Cor Image Studio Software.

### Monocyte Adhesion to Endothelial Cells

Adhesion assay was performed as described previously (Ming et al., 2012). Briefly, the THP-1 cells (a human monocytic cell line) were cultured in RPMI-1640 containing 10% heat-inactivated fetal bovine serum (FBS). For adhesion assays, the THP-1 cells were labeled with CFDA-SE (5 μmol/L) in PBS at 37°C for 8 min, and the labeling was stopped with 1 ml of heat-inactivated FBS for 1 min. The labeled monocytes (4 × 10^5^ THP-1) were then added to the HUVECs that were transduced with recombinant adenoviruses expressing shRNA and cultured under normoxic or hypoxic conditions, or treated with or without DMOG (1 mmol/L) for 24 h prior to the addition of labeled monocytes. After incubation for 15 min at 37°C, the non-adherent THP-1 cells were washed twice with PBS and fixed in 2% paraformaldehyde. The images of adherent monocytes were taken under the fluorescent microscope (five different fields per sample were captured). The number of adherent monocytes was counted using the Image J software (U.S. National Institutes of Health).

### Detection of Mitochondrial Superoxide

**Figure 1:**
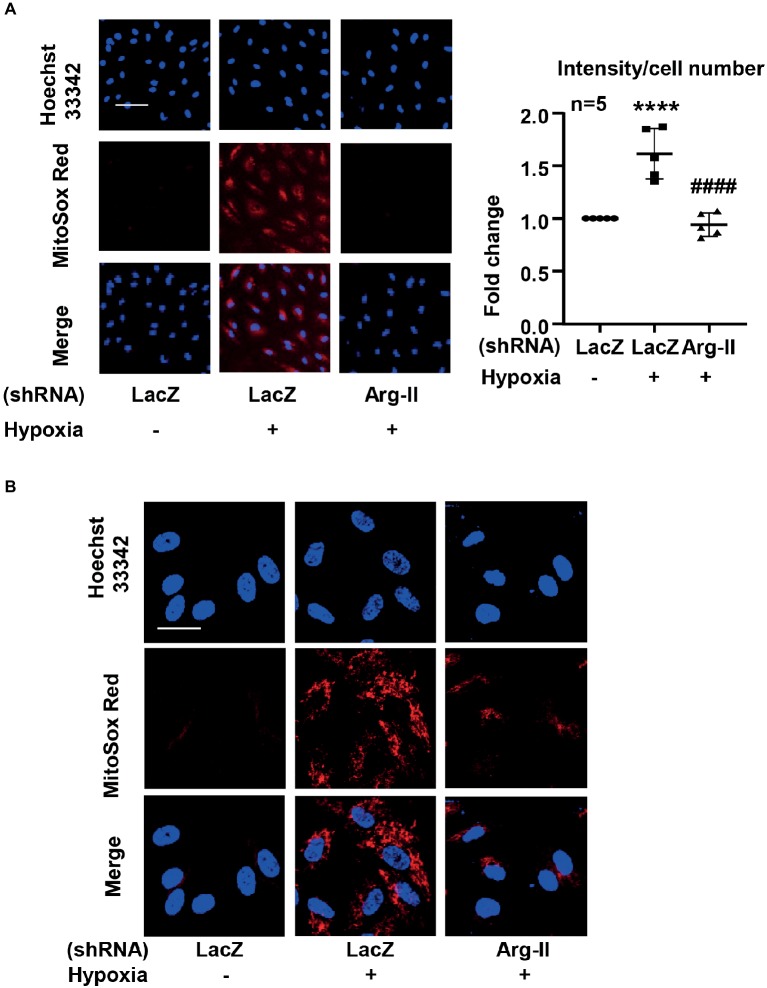
Hypoxia increased mitochondrial superoxide production in HUVECs *via* inducing Arg-II protein level. The cells were transduced with rAd/U6-LacZ^shRNA^ as control or rAd/U6-Arg-II^shRNA^ to silence Arg-II gene, and then exposed to normoxia or hypoxia for 48 h. **(A)** Cells were then subjected to mitochondrial superoxide detection with MitoSox (red) followed by nuclei staining with Hoechst 33342 (blue). The images were taken through 10 × objectives with Zeiss fluorescence microscope directly after MitoSox staining without a post-staining fixation step. Scale bar = 50 μm. The merged images are also shown. The graph shows the quantification of the signals. Data are presented as mean ± SD. ^****^p < 0.0001 vs. corresponding shRNA-LacZ cells exposed to normoxia, ^####^*p* < 0.0001 vs. shRNA-LacZ cells exposed to hypoxia. **(B)** Merged images of a higher resolution and magnification revealing the mitochondrial localization of the MitoSox signal. The images were taken from MitoSox staining with a post-staining fixation step through 40 × objectives with Leica TCS SP5 confocal laser microscope. Scale bar = 30 μm.

Mitochondrial superoxide detection was performed by using MitoSox as described previously (Ming et al., 2012; Chavan et al., 2017). Briefly, the cells were incubated with MitoSox (5 μmol/L) for 10 min. After washing, the cells were counterstained with Hoechst 33342. Images were taken through 10 × objectives with Zeiss fluorescence microscope. The intensity of the fluorescence was quantified by Image J software and normalized by cell number. For visualizing the mitochondrial localization of the MitoSox signal, after staining with MitoSox and washing, the cells were fixed with 3.7% of paraformaldehyde followed by counterstaining with Hoechst 33342 and then subjected to imaging through 40 × objectives with Leica TCS SP5 confocal laser microscope. Of note, the staining without a post-staining fixation step (for imaging using Zeiss fluorescence microscope shown in Figure 1A) shows similar results as that obtained from staining with a post-staining fixation step (for imaging using confocal microscope shown in Figure 1B), providing evidence that the post-staining fixation procedure does not introduce artifacts that may affect the results.

### Statistical Analysis

In all experiments, *n* indicates the number of independent experiments. The Kolmogorov–Smirnov test was used to first determine whether the data deviate from Gaussian distributions. Since all data are normally distributed, statistical analysis was performed with the Student’s *t*-test for unpaired observations or ANOVA with Bonferroni’s post-test, and data are expressed as mean ± SD. Differences were considered statistically significant at *p* < 0.05.

## Results

### Hypoxia Increases Arginase Type II Protein Level in Endothelial Cells

**Figure 2:**
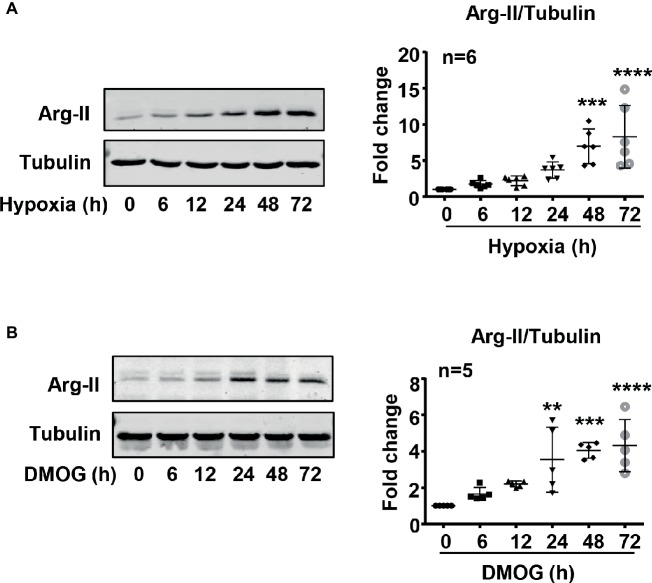
Hypoxia and DMOG enhanced Arg-II protein level in HUVECs. **(A)** HUVECs were cultured under hypoxic condition (0.2% O_2_) for indicated time, with matched control under normoxic condition. Immunoblotting shows hypoxia enhances the protein level of Arg-II. **(B)** The cells were treated with control medium or 1 mmol/L DMOG for indicated time under normoxic condition. Immunoblotting indicates that DMOG increases Arg-II protein level. The graphs on the right show the quantification of the signals on immunoblots. Data are presented as mean ± SD. One-way ANOVA analyzing differences between each treatment group and the control group. ^**^p < 0.01, ^***^p < 0.001, ^****^p < 0.0001 vs. control group.

To study the effect of hypoxia on Arg-II protein level in HUVECs, the cells were cultured under **normoxic** and hypoxic conditions for 6–72 h. The protein level of Arg-II in HUVECs was determined by immunoblotting. As shown in Figure 2A, hypoxia significantly enhanced Arg-II protein levels in a time-dependent manner. The Arg-II protein level was also enhanced in the cells treated with DMOG (Figure 2B), an agent that mimics hypoxic condition by inhibiting activity of prolyl-4-hydroxylase, resulting in HIFα accumulation. The results suggest a role of HIF in upregulation of Arg-II protein level in the endothelial cells under hypoxic condition.

### Hypoxia-Inducible Factor 1α Mediates Arginase Type II Upregulation Induced by Hypoxia

**Figure 3:**
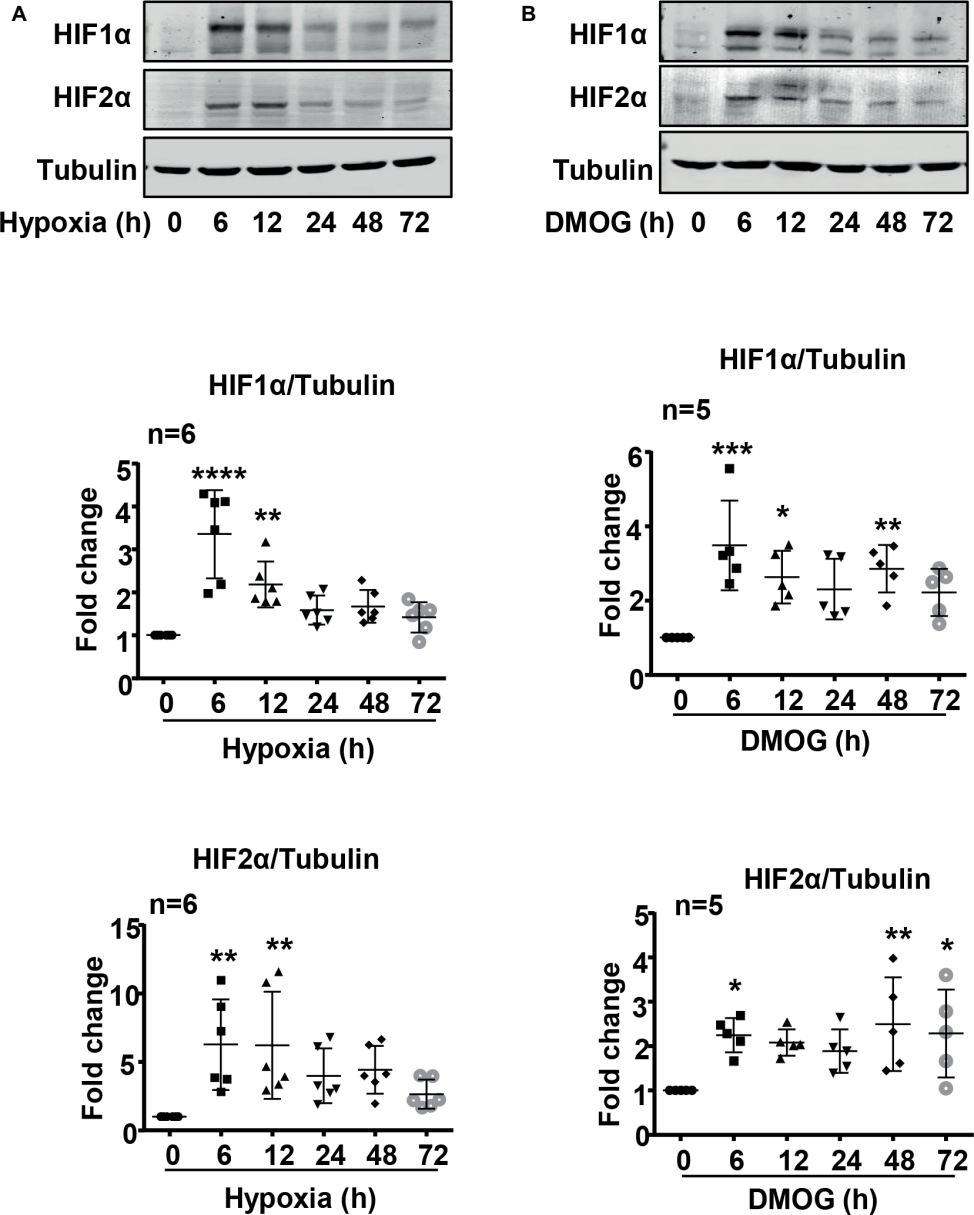
Hypoxia and DMOG upregulated HIF1α and HIF2α levels in HUVECs. **(A)** The cells were exposed to normoxia or hypoxia for indicated time. Exposure to hypoxia enhanced HIF1α and HIF2α protein level examined by immunoblotting. **(B)** The cells were treated with control medium or 1 mmol/L DMOG for indicated time. Treatment of the HUVECs with DMOG increases HIF1α and HIF2α protein level detected by immunoblotting. The graphs on the right show the quantification of the signals on immunoblots. Data are presented as mean ± SD. One-way ANOVA analyzing differences between each treatment group and the control group. ^*^p < 0.05, ^**^p < 0.01, ^***^p < 0.001, ^****^p < 0.0001 vs. control group.

Indeed, both HIF1α and HIF2α levels were upregulated over 72 h in the endothelial cells under hypoxic condition (Figure 3). The maximum elevation of HIFs occurred at 6 h of hypoxia and remained at significantly higher levels as compared with the normoxia controls (Figure 3A). As expected, DMOG treatment exerted similar effects on HIF levels in the cells (Figure 3B).

**Figure 4:**
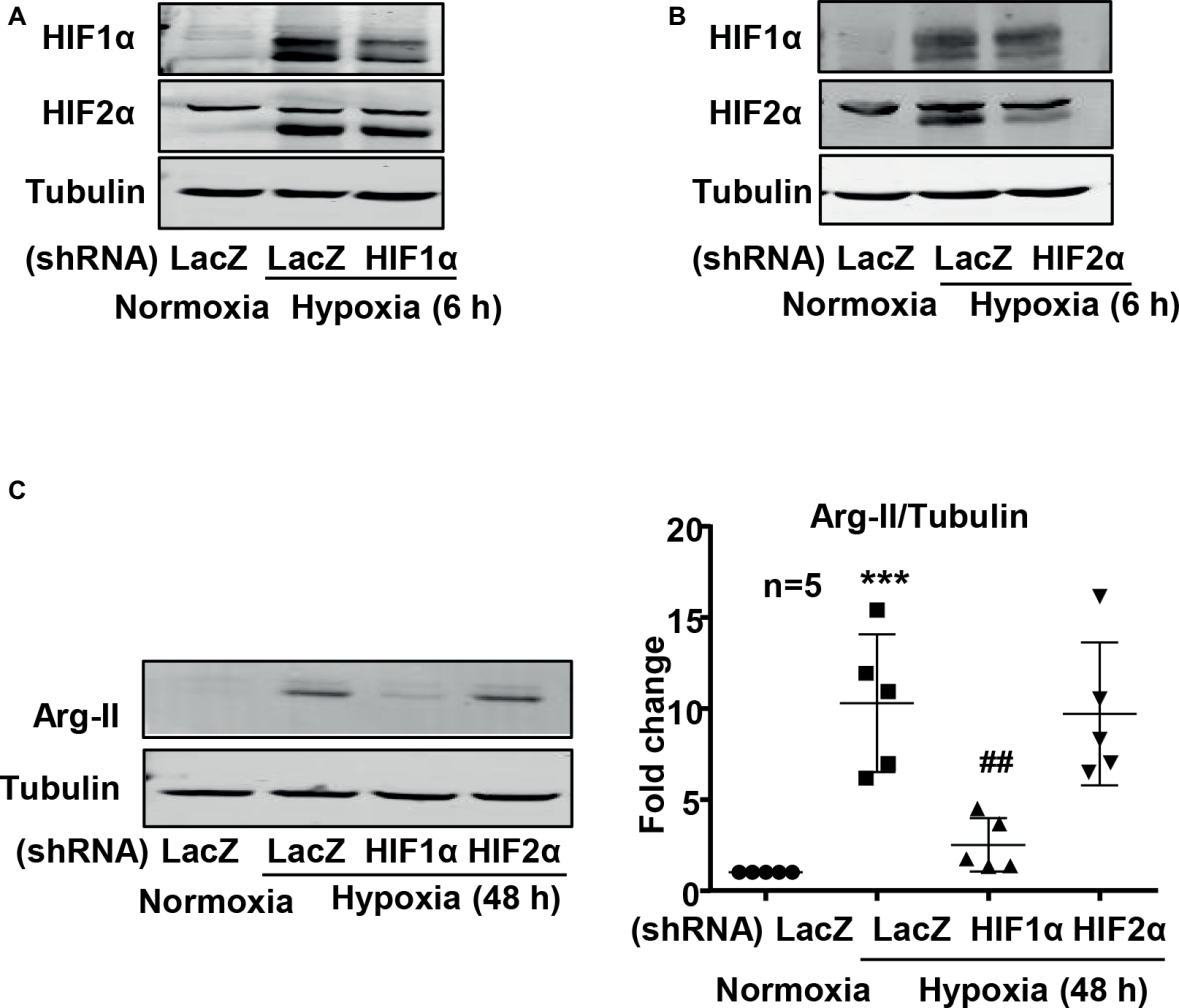
Silencing HIF1α prevented hypoxia-induced upregulation of Arg-II in HUVECs. HUVECs were transduced with rAd/U6-LacZ^shRNA^ as control or rAd/U6-HIF1α^shRNA^ and rAd/U6-HIF2α^shRNA^ to silence HIF1α and HIF2α gene, respectively. **(A)** Immunoblotting analysis confirms HIF1α silencing. **(B)** Immunoblotting analysis validates HIF2α silencing. **(C)** Immunoblotting shows silencing HIF1α, but not HIF2α, inhibited Arg-II upregulation in HUVECs induced by hypoxia. Graph on the right presents the quantification of the signals on immunoblots. Data are presented as mean ± SD. ^***^p < 0.001 vs. corresponding shRNA-LacZ cells cultured under normoxic condition, ^##^*p* < 0.01 vs. shRNA-LacZ cells incubated under hypoxic condition.

Next, the cells were transduced with recombinant adenovirus (rAd) carrying shRNA against human HIF1α (rAd/U6-HIF1α^shRNA^) or HIF2α (rAd/U6-HIF2α^shRNA^) and were subjected to either normoxic or hypoxic condition. The specificity and efficiency of HIF1α and HIF2α silencing were confirmed by immunoblotting (Figures 4A,B). Interestingly, silencing HIF1α, but not HIF2α, prevented Arg-II upregulation in cells under hypoxic condition (Figure 4C). These results demonstrate that hypoxia-induced upregulation of Arg-II in endothelial cells was mediated by HIF1α, but not HIF2α.

### Hypoxia-Induced Intercellular Adhesion Molecule 1 Upregulation in Endothelial Cells Is Mediated by Arginase Type II

**Figure 5:**
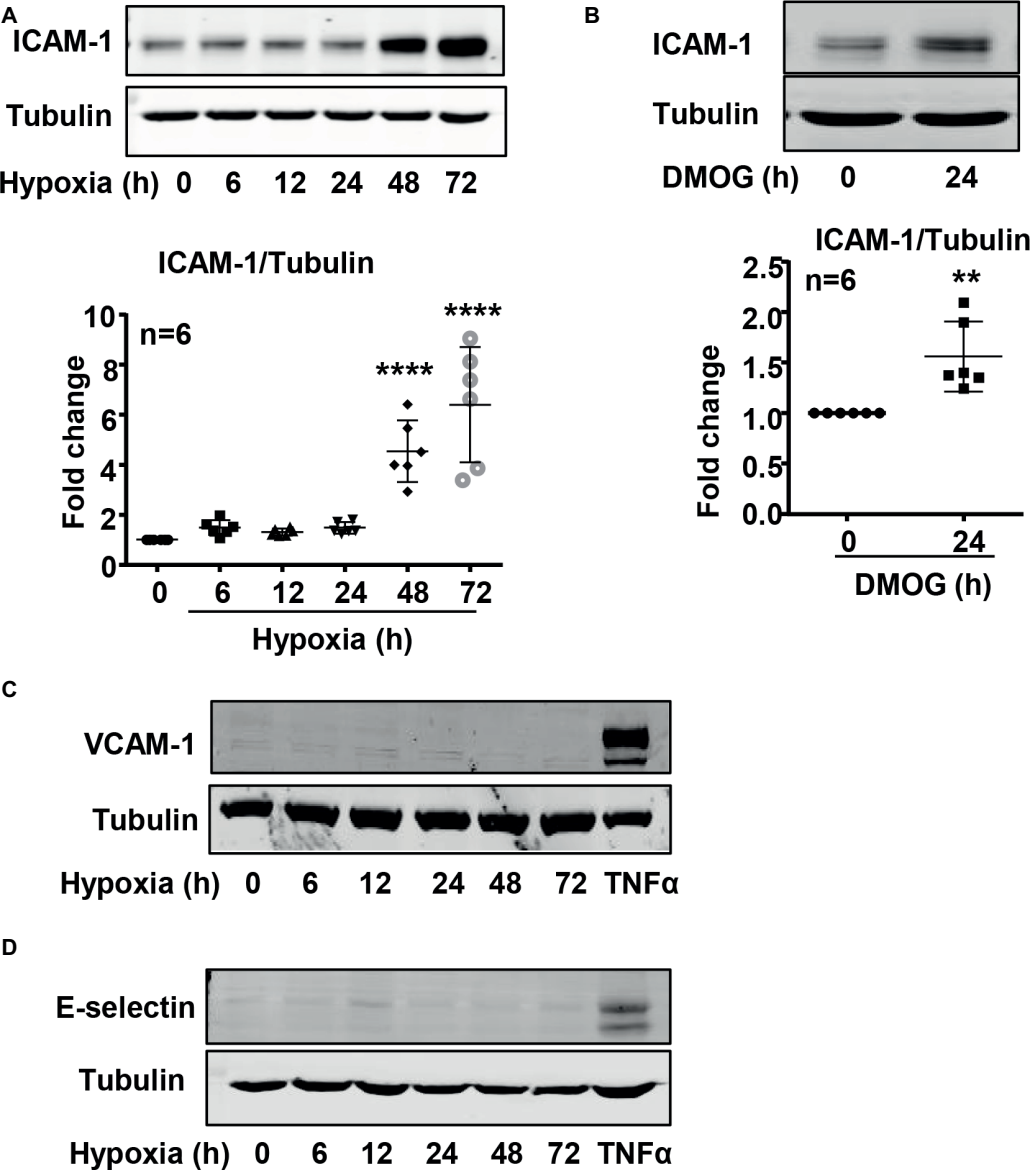
Hypoxia and DMOG increased ICAM-1 protein level in HUVECs. **(A,C,D)** HUVECs were incubated under normoxic or hypoxic condition (0.2% O_2_) for indicated time. Immunoblotting shows hypoxia enhances ICAM-1 protein level **(A)**, but not VCAM-1 (C) and E-selectin **(D)** protein level. **(B)** The cells were treated with control medium or 1 mmol/L DMOG for 24 h under normoxic condition. Immunoblotting reveals that DMOG increases ICAM-1 protein level. Quantification of the signals on immunoblots is presented as graphs below. Data are presented as mean ± SD. One-way ANOVA analyzing differences between each treatment group and the control group **(A)** and *t*-test **(B)**. ^**^p < 0.01, ^****^p < 0.0001 vs. control group.

**Figure 6:**
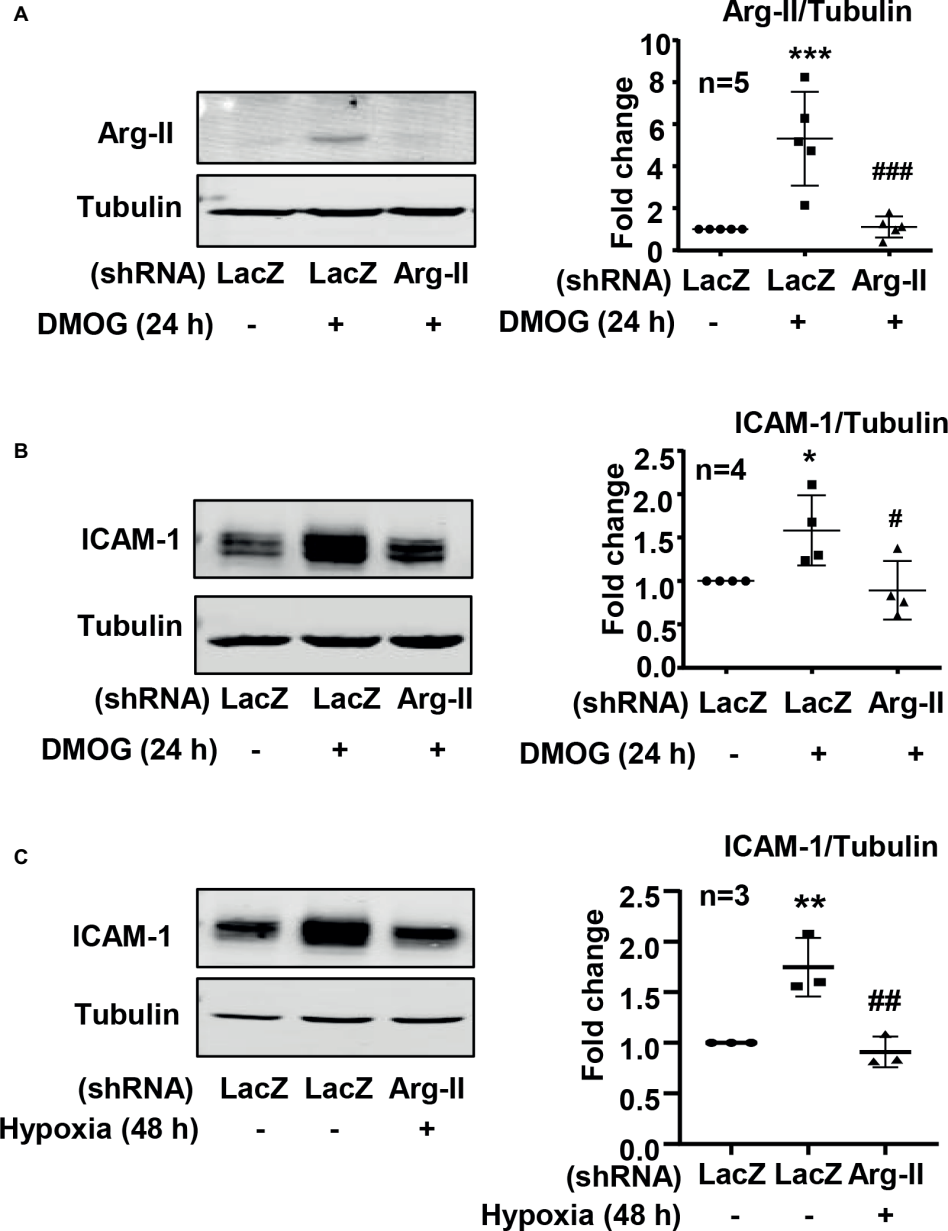
Silencing Arg-II in HUVECs prevented the ICAM-1 upregulation induced by hypoxia or DMOG. The cells were transduced with rAd/U6-LacZ^shRNA^ as control or rAd/U6-Arg-II^shRNA^ to silence Arg-II gene, and then treated with or without DMOG (1 mmol/L) for 24 h, or incubated under normoxic or hypoxic condition for 48 h. **(A)** Immunoblotting shows Arg-II silencing in HUVECs. **(B)** Silencing Arg-II in HUVECs reverses DMOG-induced increase in protein level of ICAM-1 analyzed by immunoblotting. **(C)** Silencing Arg-II abrogates hypoxia-induced increase in ICAM-1 level measured by immunoblotting. The plot graphs on the right present the quantification of the signals on immunoblots. Data are presented as mean ± SD. ^*^p < 0.05, ^**^p < 0.01, ^***^p < 0.001 vs. corresponding shRNA-LacZ group, ^#^*p* < 0.05, ^##^*p* < 0.01, ^###^*p* < 0.001 vs. shRNA-LacZ cells treated with DMOG or cultured in the hypoxic condition.

It is well recognized that increased endothelial adhesion molecule expression results in enhanced adhesion and transmigration of monocytes into the vascular wall. We examined whether hypoxia induces protein level of adhesion molecules such as VCAM-1, ICAM-1, and E-selectin in endothelial cells. The protein level of ICAM-1 was enhanced in endothelial cells under the hypoxic condition over 72 h in a time-dependent manner (Figure 5A). Furthermore, treatment of the cells with DMOG also enhanced ICAM-1 protein level in the cells (Figure 5B). However, hypoxia did not induce VCAM-1 and E-selectin protein level in the endothelial cells (Figures 5C,D). Importantly, silencing Arg-II in the endothelial cells as validated by immunoblotting (Figure 6A) significantly reduced ICAM-1 upregulation by DMOG (Figure 6B). Similarly, protein level of ICAM-1 under hypoxic condition was significantly reduced in cells in which Arg-II gene was silenced (Figure 6C). These results suggest that hypoxia-induced upregulation of ICAM-1 in endothelial cells is mediated by Arg-II.

### Silencing Arginase Type II in Endothelial Cells Inhibits Hypoxia-Induced Monocyte-Endothelial Cell Interaction

**Figure 7:**
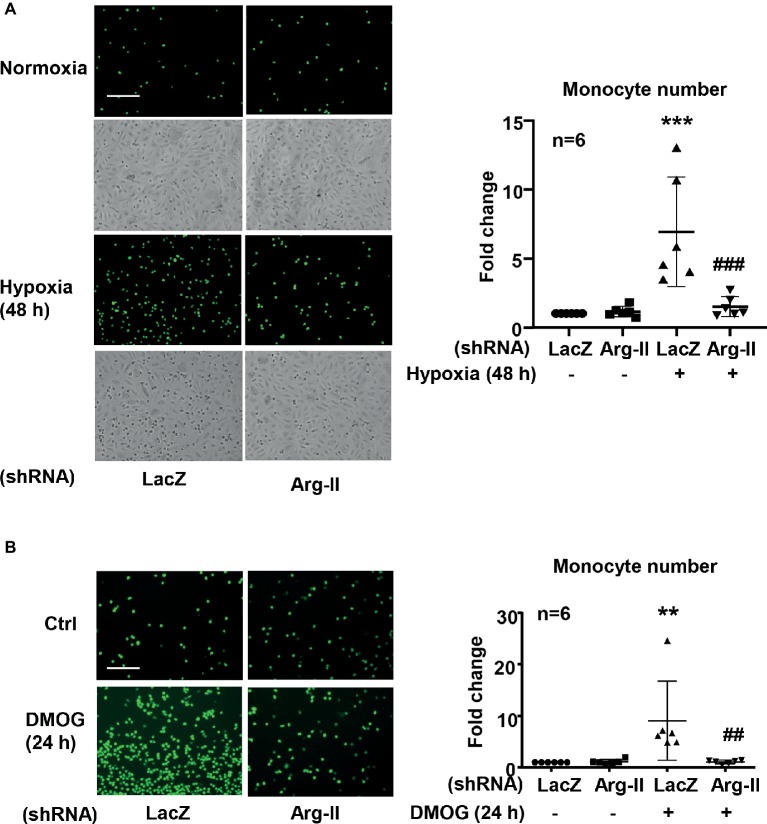
Silencing Arg-II in HUVECs inhibited Hypoxia **(A)**- and DMOG **(B)**-enhanced monocyte adhesion. CFDA-SE fluorescence labeled THP-1 monocyte adhesion to endothelial cells that were transduced with shRNA as indicated. The phase contrast images reveal the similar plating density of endothelial cells. The plot graph shows quantifications of the adhered monocytes. Data are presented as mean ± SD. ^**^p < 0.01, ^***^p < 0.001 vs. corresponding shRNA-LacZ group, ^##^*p* < 0.01, ^###^*p* < 0.001 vs. shRNA-LacZ cells treated with DMOG. Scale bar = 100 μm.

As a result of increased ICAM-1 protein level in endothelial cells under hypoxic condition, an increase in monocyte adhesion to the endothelial cells was observed. Indeed, in endothelial cells exposed to hypoxic condition, monocyte adhesion to the endothelial cells was significantly enhanced, which was inhibited by Arg-II silencing (Figure 7A). Of note, the phase contrast images reveal the similar plating density of endothelial cells. Similar results were obtained with DMOG treatment. DMOG significantly enhanced monocyte adhesion to endothelial cells, which was prevented by Arg-II silencing (Figure 7B). These findings indicate that hypoxia-induced monocyte adhesion to endothelial cells was mediated by endothelial Arg-II.

### Hypoxia Increases Intercellular Adhesion Molecule 1 Protein Level Through Arginase Type II and Mitochondrial Reactive Oxygen Species in Endothelial Cells

**Figure 8:**
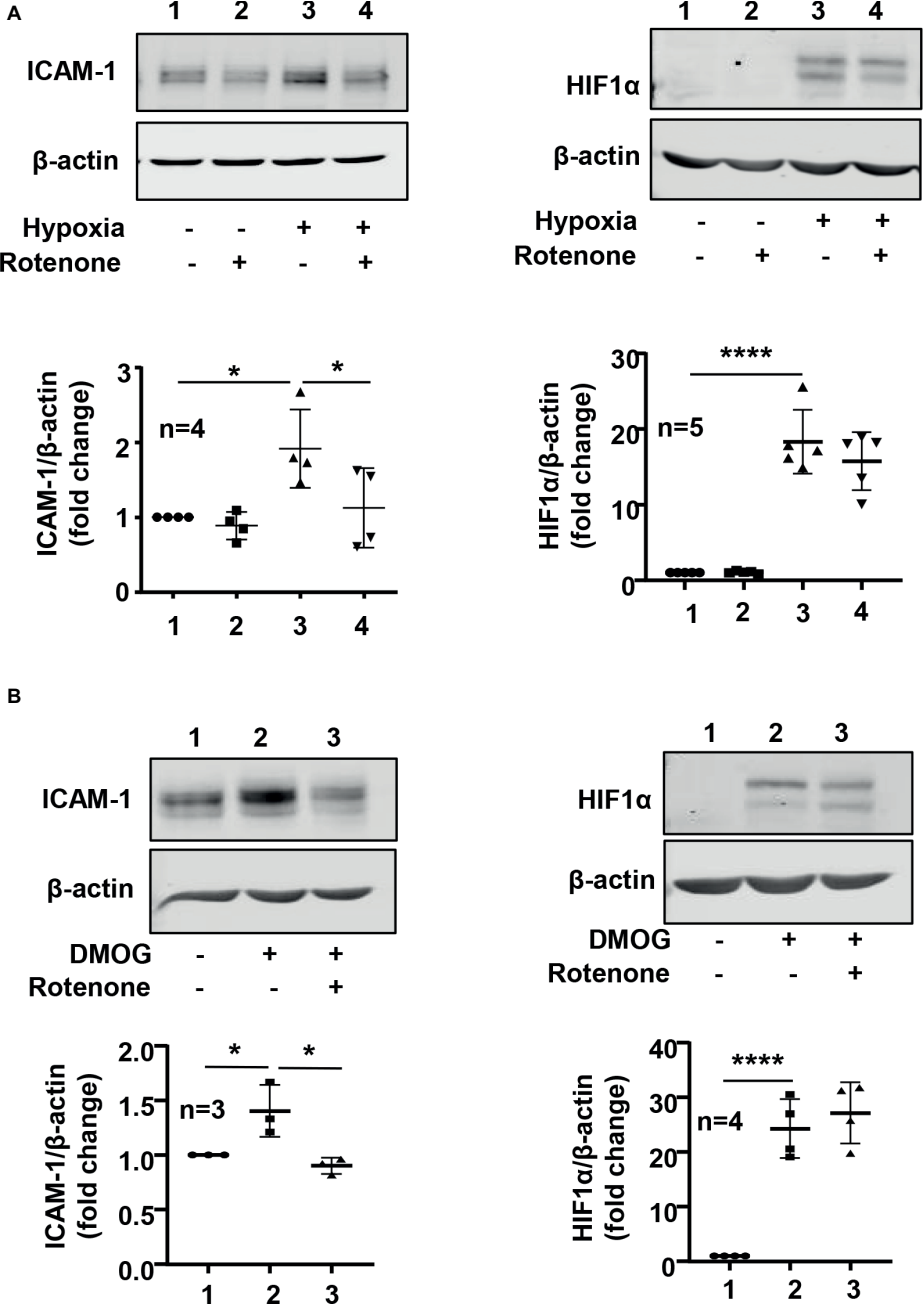
Mitochondrial electron transport inhibitor rotenone reversed hypoxia- and -DMOG-induced increase in ICAM-1 protein level without affecting HIF1α level in HUVECs. The cells were pretreated with or without rotenone (2 μmol/L) for 1 h and then incubated under normoxic or hypoxic condition **(A)** or treated with DMOG **(B)** for 48 h **(A)** or 24 h **(B)**, respectively. The protein level of ICAM-1 and HIF1α in HUVECs was detected by immunoblotting. The graph on the right indicates the quantification of the signals. Data are showed as mean ± SD. ^*^p < 0.05, ^****^p < 0.0001 between indicated groups.

We further investigated roles of Arg-II in mitochondrial dysfunction and ICAM-1 protein level under hypoxic condition. As shown in Figure 1, hypoxia increased mitochondrial reactive oxygen species (ROS) production, which was prevented by silencing Arg-II in the endothelial cells, suggesting that hypoxia enhances mitochondrial ROS production through Arg-II in the cells. Furthermore, inhibition of mitochondrial respiration complex-I by rotenone prevented increase in ICAM-1 without significant effect on HIF1α under hypoxic condition (Figure 8A). Similar results were obtained for DMOG-induced ICMA-1 and HIF1α (Figure 8B).

## Discussion

Our current study confirms the previous findings that hypoxia is a potent stimulus for Arg-II protein level in endothelial cells (Krotova et al., 2010; Prieto et al., 2011; Cowburn et al., 2016; Pandey et al., 2018). Furthermore, we provide evidence that this effect of hypoxia enhances monocyte-endothelium interaction due to increased ICAM-1 protein level which is mediated by HIF1α-Arg-II-mitochondrial ROS in endothelial cells exposed to hypoxia.

Under hypoxic condition, cellular adaptation oxygen-sensing pathways are activated, which relies on the transcription factor HIF. HIF is a dimeric protein complex composed of alpha and beta subunits. Under normoxic conditions, HIF1α and HIF2α are hydroxylated by the oxygen-dependent prolyl-hydrolase (PHD) leading to ubiquitination of HIF1α and HIF2α by the von Hippel–Lindau protein (VHL) E3 ubiquitin ligase and their subsequent rapid degradation (Semenza, 2014). Under hypoxic conditions, HIF-PHD interaction is inhibited, resulting in stabilization and accumulation of alpha subunit of HIFs, allowing transcription of hypoxia-driven target genes (Semenza, 2014). Arg-II has been demonstrated to be one of the HIF-regulated genes (Krotova et al., 2010; Prieto et al., 2011; Mimura et al., 2012; Cowburn et al., 2016; Pandey et al., 2018). In our current study, we show that Arg-II upregulation in human endothelial cells is mediated by HIF1α, since hypoxia- and DMOG-induced Arg-II protein level is inhibited upon silencing HIF1α but not HIF2α. This is in line with the genome-wide analysis of HIF1-binding sites by ChIP-seq in endothelial cells which reveals that Arg-II gene intron 2 has HIF1-binding regions (Mimura et al., 2012). It is, however, in contrast to previous studies showing that the hypoxia-induced upregulation of Arg-II is mediated through HIF2α in pulmonary endothelial cells (Krotova et al., 2010; Cowburn et al., 2016). The discrepancy of Arg-II upregulation through HIF1 in HUVEC in our current study or through HIF2 in pulmonary endothelial cells in previous studies (Krotova et al., 2010; Cowburn et al., 2016) may be due to different origin of endothelial cells. The transcription factor Kruppel-like factor 15 (KLF15) has recently been shown to bind directly to the Arg-II promoter and repress endothelial Arg-II expression (Pandey et al., 2018). Interestingly, KLF15 was decreased and binding of KLF15 to the Arg-II promoter was relieved in response to hypoxia. Moreover, overexpression of KLF15 reversed hypoxia-induced augmentation of arginase activity in human pulmonary microvascular endothelial cells (HPMECs), suggesting that KLF15 is a critical regulator of Arg-II transcription in response to hypoxia in HPMEC (Pandey et al., 2018). Whether KLF15 contributes to the Arg-II upregulation in HUVEC remains elusive.

Hypoxia is associated with inflammations (Eltzschig and Carmeliet, 2011; Abe et al., 2017). Studies showed that hypoxia induces endothelial activation with enhanced endothelial adhesion molecule expression (Yoon et al., 2006; Ben-Shoshan et al., 2009). Our present study confirmed upregulation of ICAM-1 by both hypoxia and **hypoxia mimetic DMOG**. VCAM-1 and E-selectin level is not affected by hypoxia. We further demonstrate that hypoxia-induced ICAM-1 protein level is mediated through Arg-II. This conclusion is supported by the fact that the hypoxia- or DMOG-induced ICAM-1 protein level is inhibited upon silencing Arg-II. In support of this finding, hypoxia- or DMOG-induced monocyte adhesion to endothelial cells is also markedly abolished by silencing Arg-II. Since Arg-II is regulated by HIF1α, but not HIF2 α, in endothelial cells in response to hypoxia, our results suggest that hypoxia-induced endothelial activation is mediated by HIF1α which leads to Arg-II upregulation. Interestingly, studies have demonstrated that HIF1*α* is induced by pro-inflammatory stimuli lipopolysaccharide (LPS) and interferon gamma (IFNγ) and plays a critical role in pro-inflammatory M1 macrophage activation (Cramer et al., 2003; Bonello et al., 2007; Rius et al., 2008), whereas HIF2α is induced by anti-inflammatory stimuli IL-4 and IL-13 and expressed exclusively in anti-inflammatory M2 macrophage (Takeda et al., 2010). Our data thus support a role ofHIF1α in pro-inflammatory response in endothelial cells in addition to that in M1 macrophages, and for the first time characterized Arg-II as a downstream mediator of HIF1α in the pro-inflammatory response.

Finally, we provide evidence that HIF1α-Arg-II-mediated endothelial ICAM-1 upregulation under hypoxic condition involves mitochondrial ROS generation, since hypoxia-induced mitochondrial ROS generation is suppressed by Arg-II silencing and inhibition of mitochondrial ROS blocks hypoxia-induced ICAM-1. It has been reported that hypoxia-induced ROS production is mainly from mitochondria (Pearlstein et al., 2002), which is in line with our finding. Moreover, in supporting our finding of HIF1α-Arg-II-mitochondrial ROS cascade as a mechanism of hypoxia-induced endothelial ICAM-I protein level, previous studies have demonstrated that mitochondrial ROS increases ICAM-1 through AP-1 and NF-kB (Hawkins et al., 2007; Li et al., 2016). Furthermore, increased ROS generation has been shown to be associated with leukocyte-endothelial cell adhesion (Wood et al., 2000). The fact that rotenone, an inhibitor of mitochondrial respiration chain, blocks hypoxia-induced ICAM-1 protein level induced by Arg-II suggests that Arg-II promotes mitochondrial ROS through affecting mitochondrial respiration chain. In contrast to the previous report showing that mitochondrial ROS stabilizes HIF1α during hypoxia (Chandel et al., 2000), inhibition of mitochondrial ROS with rotenone did not affect HIF1α level either under hypoxic condition or under condition of DMOG treatment, indicating that mitochondrial ROS does not play a major role in regulation of HIF1α levels under our experimental condition. How Arg-II perturbs mitochondrial respiration chain requires further investigation.

## Conclusion

**Figure 9:**
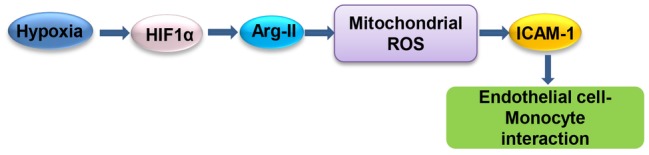
Schematic summary of the current study. Hypoxia enhances ICAM-1 protein level and monocyte-endothelial interaction through HIF1α-Arg-II-mitochondrial ROS.

Our study demonstrates that hypoxia enhances endothelial ICAM-1 protein level and monocyte-endothelium interaction through HIF1α-Arg-II-mitochondrial ROS (Figure 9). Our study reveals an important molecular and cellular mechanism of hypoxia-induced endothelial dysfunction and suggests that targeting HIF1α-Arg-II-mitochondrial ROS cascade could be a promising therapeutic strategy to prevent hypoxia-induced vascular damage/dysfunction.

## Data Availability

The raw data supporting the conclusions of this manuscript will be made available by the authors, without undue reservation, to any qualified researcher.

## Author Contributions

XL and PA contributed to acquisition, analysis, or interpretation of data for the work; contributed to preparation of figures; and critically revised the manuscript for important intellectual content. X-FM and ZY contributed to design of the work; analyzed and interpreted primary research papers; drafted the manuscript; and critically revised the manuscript for important intellectual content. All authors agree to be accountable for the content of the work.

### Conflict of Interest Statement

The authors declare that the research was conducted in the absence of any commercial or financial relationships that could be construed as a potential conflict of interest.
